# Abiotic and biotic factors jointly influence the contact and environmental transmission of a generalist pathogen

**DOI:** 10.1002/ece3.70167

**Published:** 2024-08-16

**Authors:** Daniel C. Suh, Stacey L. Lance, Andrew W. Park

**Affiliations:** ^1^ Odum School of Ecology University of Georgia Athens Georgia USA; ^2^ Center for the Ecology of Infectious Diseases University of Georgia Athens Georgia USA; ^3^ Savannah River Ecology Laboratory University of Georgia Aiken South Carolina USA; ^4^ Department of Infectious Diseases, College of Veterinary Medicine University of Georgia Athens Georgia USA

**Keywords:** abundance, amphibians, community competence, diversity–disease relationships, ranavirus, temperature, wetlands

## Abstract

The joint influence of abiotic and biotic factors is important for understanding the transmission of generalist pathogens. Abiotic factors such as temperature can directly influence pathogen persistence in the environment and will also affect biotic factors, such as host community composition and abundance. At intermediate spatial scales, the effects of temperature, community composition, and host abundance are expected to contribute to generalist pathogen transmission. We use a simple transmission model to explain and predict how host community composition, host abundance, and environmental pathogen persistence times can independently and jointly influence transmission. Our transmission model clarifies how abiotic and biotic factors can synergistically support the transmission of a pathogen. The empirical data show that high community competence, high abundance, and low temperatures correlate with high levels of transmission of ranavirus in larval amphibian communities. Discrete wetlands inhabited by larval amphibians in the presence of ranavirus provide a compelling case study comprising distinct host communities at a spatial scale anticipated to demonstrate abiotic and biotic influence on transmission. We use these host communities to observe phenomena demonstrated in our theoretical model. These findings emphasize the importance of considering both abiotic and biotic factors, and concomitant direct and indirect mechanisms, in the study of pathogen transmission and should extend to other generalist pathogens with the capacity for environmental transmission.

## Graphical abstract

Host community composition and abundance and environmental conditions such as ambient temperature all influence disease transmission but are often considered independently. We use a theoretical model to study the joint effects of these factors on the transmission of a generalist pathogen with capacity for contact and environmental transmission, and then we compare these results to empirical observations of ranavirus transmission in larval amphibian communities to show that these factors jointly influence transmission.

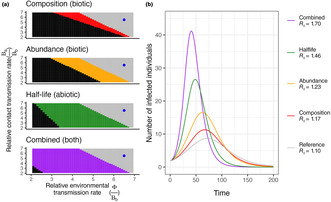

## 1 INTRODUCTION

Environmental conditions have direct and indirect effects on pathogen transmission. Directly, abiotic factors, including temperature and humidity, can influence transmission via multiple mechanisms, such as by altering persistence times outside of their host (Gray et al., 2009; Nazir et al., 2012). Indirectly, the environment alters transmission by affecting host community composition, impacting host availability (Love et al., 2016). For example, temperature can affect host growth rates and population sizes, resulting in changes in the absolute abundance of susceptible hosts (Savage et al., 2004; Sibly & Hone, 2002) and the relative abundance of different host species in a community (Altizer et al., 2013; Blaustein et al., 2010). Over time and space, environmental conditions vary naturally, resulting in altered transmission potential. Despite the importance of both biotic and abiotic factors in pathogen transmission, the existing diversity–disease literature does not commonly address both together. Instead, studies at the local and regional scale typically focus on biotic factors (Johnson et al., 2015; Rohr et al., 2019) while abiotic factors are more often considered at larger spatial scales (Cohen et al., 2016), emphasizing the need for studies at an intermediate spatial scale where multiple communities of hosts are linked by dispersal.

Separate lines of evidence suggest that the abundance of hosts, the composition of host communities, and the direct effects of environmental conditions influence pathogen transmission. Pathogens with density‐dependent transmission rely on host species abundance to invade and persist within a host population (Fenton et al., 2002; Hopkins et al., 2020; Patterson & Ruckstuhl, 2013). For generalist pathogens, variation in host competence, the ability of a host to acquire and transmit a pathogen, across species determines transmission potential (Martin et al., 2016). Recently, competence has been extended to consider how it scales across host species, populations, and communities (Downs et al., 2019; Johnson et al., 2013). Community competence, for example, the average probability that an individual animal in a multispecies community becomes infected presents important summary information on how prone a host community may be for a disease outbreak (Mihaljevic et al., 2014). This attribute is strongly determined by the relative abundance of host species (Johnson et al., 2013). It remains an open question how competence varies across a host phylogeny, and whether that same phylogeny influences community structure, either through competition or environmental filtering. Consequently, joint consideration of how host phylogenies influence competence and relative abundance may help explain disease outbreaks in host communities.

Further, pathogens with the capacity for environmental transmission are subject to their surrounding environmental conditions. For example, the persistence of influenza virus in the environment can enhance transmissibility, and persistence times are reduced under acidic conditions, warmer temperatures, and high salinity (Brown et al., 2009; Rohani et al., 2009; Sooryanarain & Elankumaran, 2015). However, while studies tend to focus on either host abundance, community composition, or environmental conditions, these factors jointly influence transmission and are non‐independent. Host abundance and community structure often fluctuate in response to environmental conditions (Werner et al., 2007), and these changes in community structure can result in dramatic shifts in community competence (Streicker et al., 2013). Over seasonal timescales, species exhibit distinct phenologies and experience dynamic strengths of competition (Rudolf, 2019), which can simultaneously alter both community structure and size. Consequently, the separate and combined effects of host community composition, host abundance, and environmental conditions can improve our understanding of how generalist pathogens invade and persist within variable host communities (Becker et al., 2012; Johnson & Brunner, 2014; Nazir et al., 2012). Our study contributes to this effort by generating mechanistic insight into diversity–disease patterns, moving beyond the more commonly observed research that relies on patterns of host species richness and infection prevalence in communities (Rohr et al., 2019).

It is likely that many infectious disease systems are under the strong influence of abiotic and biotic factors. Here, we use ranavirus infection data in larval amphibian communities to illustrate that the joint influence of biotic and abiotic drivers of transmission is at play. Many pathogens utilize multiple host species and exhibit environmental transmission, including wildlife and livestock pathogens, such as foot‐and‐mouth disease virus (FMDV) and rotaviruses (Kraay et al., 2018; Miguel et al., 2013). In some cases, these pathogens are also capable of zoonotic transmission with direct implications for human health (Cook, 2004; Martella et al., 2010). Furthermore, recent reports of avian influenza transmission in mammals highlight the need for understanding the transmission of multihost, multimodal pathogens (Agüero et al., 2023; Plaza et al., 2024; Puryear et al., 2023; Rohani et al., 2009). In systems such as these, the availability of hosts and environmental conditions will influence pathogen transmission due to the pathogen's capacity for both environmental and contact transmission. However, many of these studies are limited to an anthropocentric scope and rarely consider the diversity–disease dynamics in wildlife populations, potentially overlooking consequential promoters of transmission (Brnić et al., 2022; Miguel et al., 2013). Conversely, studies of wildlife pathogens, such as ranaviruses, often include explicit consideration of the community of host species available. All these systems can benefit from a deeper understanding of the joint biotic and abiotic factors involved and how different modes of transmission can be favored under changing environmental or host conditions.

Ranaviruses represent a genus of viruses known to be associated with global amphibian declines and exhibit both contact and environmental transmission (Brunner et al., 2017; Sage et al., 2019). Ranaviruses in larval amphibian communities are useful for studying the effects of host community composition on transmission potential because there is a large variation in competence across host species, and the composition and abundance of host communities change over space and time (Love et al., 2016; Snyder et al., 2023). Abiotic factors, namely, temperature, influence community composition and directly influence environmental transmission rates; environmental persistence of the virus is sensitive to abiotic factors. For example, degradation rates are highest under warmer water temperatures (Brunner & Yarber, 2018; Nazir et al., 2012).

To establish how biotic and abiotic factors jointly influence transmission, we developed a mechanistic model that incorporates the direct effects of the environment on the pathogen, specifically the environmental persistence time, as well as changes in both host abundance and community composition. While important theoretical developments have described transmission in multihost communities (Dobson, 2004; Fountain‐Jones et al., 2018; Holt et al., 2003; Roche et al., 2012), and via multiple transmission modes (Eisenberg et al., 2013; Majewska et al., 2019; Rohani et al., 2009), their joint consideration in models is lacking. Accordingly, we develop such a model and assess the effects of host abundance, community composition, and environmental persistence on *R*_0_, the basic reproductive number for the pathogen, under a range of plausible conditions. We compare findings from the model to the empirical data on ranaviruses to demonstrate that each factor can contribute substantially to transmission and can do so simultaneously.

We contend that studying both biotic and abiotic factors, including their influence on each other, can aid in predicting the location and timing of outbreaks of generalist pathogens that employ multiple transmission modes. Our study is well poised to illustrate this phenomenon because larval amphibians occupy discrete wetlands, linked via adult movement, to form a metacommunity occurring at an intermediate spatial scale, which potentially renders biotic and abiotic factors of equal importance (Rohr et al., 2019). Further, by developing a theoretical model for understanding these joint effects, we present mechanistic insights to explain empirical patterns in our study, which are likely to apply to studies of other generalist pathogens with multiple modes of transmission as well (Bienentreu & Lesbarrères, 2020; Dillon & Meentemeyer, 2019; Youker‐Smith et al., 2018).

## 2 METHODS

### 2.1 Data collection

All empirical data in this analysis were provided by collaborators (Coleman, 2018) and had been previously collected at the United States Department of Energy's Savannah River Site (South Carolina, USA). Twenty wetlands were sampled monthly for 6 months from February to July 2016 at the Savannah River Site. Of the 120 sampling events, 96 produced data, with the others being discounted because wetlands were dry at the time of sampling. Each monthly sampling event included an estimate of larval amphibian abundance ascertained from 4 days of minnow trapping (set on day 1, checked days 2–5, closed on day 5) and 1 day of standardized dip‐net sweeps around the perimeter of the wetland. In addition to abundance, a single individual per species was collected per dip‐net sweep or minnow trap. All anuran and some caudate (newts) individuals were tested for ranavirus load using qPCR in triplicate following the general protocol described previously (Allender et al., 2013), with values averaged to determine the viral load for an individual. At the species‐level, all individuals that were analyzed for viral load were then averaged to provide a species‐level estimate of viral load, a proxy for competence. Only species that had at least three individuals tested for ranavirus were included in the analysis. Overall, over 31,000 individuals were captured and identified, 2056 were tested for ranavirus, and 334 were positive. Prevalence was estimated as the proportion of infected individuals sampled weighted by the relative abundance of that species at each sampling event (see Appendix S3). Water temperature was measured using iButton loggers (iButtonLink, LLC. Whitewater, WI, USA) placed 10 cm below the water's surface.

### 2.2 Calculation of community competence and prevalence ratio

Using species‐level competence, we calculated community competence as the weighted average of each species' competence, with weights given by the relative abundance of each species (Johnson et al., 2013). Each site‐month combination was treated as a distinct community in these calculations. We designed a metric that summarized ranavirus transmission, hereafter referred to as the prevalence ratio (*θ*), to test whether community competence, host abundance, and mean water temperature at each site‐month were correlated with ranavirus transmission as the epizootics unfolded between February and July. The prevalence ratio (*θ*) was calculated per site‐month as the percentage increase (before peak prevalence) or decrease (after peak prevalence) in prevalence during 1 month relative to the potential change in prevalence possible based on the observed peak prevalence (used on the approach to peak) or reduction to zero prevalence (used after peak), (Box 1). The prevalence ratio is advantageous because it allows us to detect whether conditions were favorable or unfavorable for the pathogen along the entire epizootic (both before and after the peak, which was typically in April to May). Before the peak, as prevalence is increasing, conditions are estimated to be favorable for the pathogen (higher *θ*) if prevalence increases appreciably. Conversely, after the peak, as prevalence is decreasing, conditions were estimated to be favorable for the pathogen if prevalence decreases minimally (again, higher *θ*). We tested if community competence, log‐transformed host abundance, and mean water temperature were significantly correlated with *θ*, using Spearman rank correlation tests with a Holm‐Bonferroni correction for multiple comparisons.

BOX 1 **Definition of the prevalence ratio metric (*θ*) and a worked example of values before and after an epizootic peak**

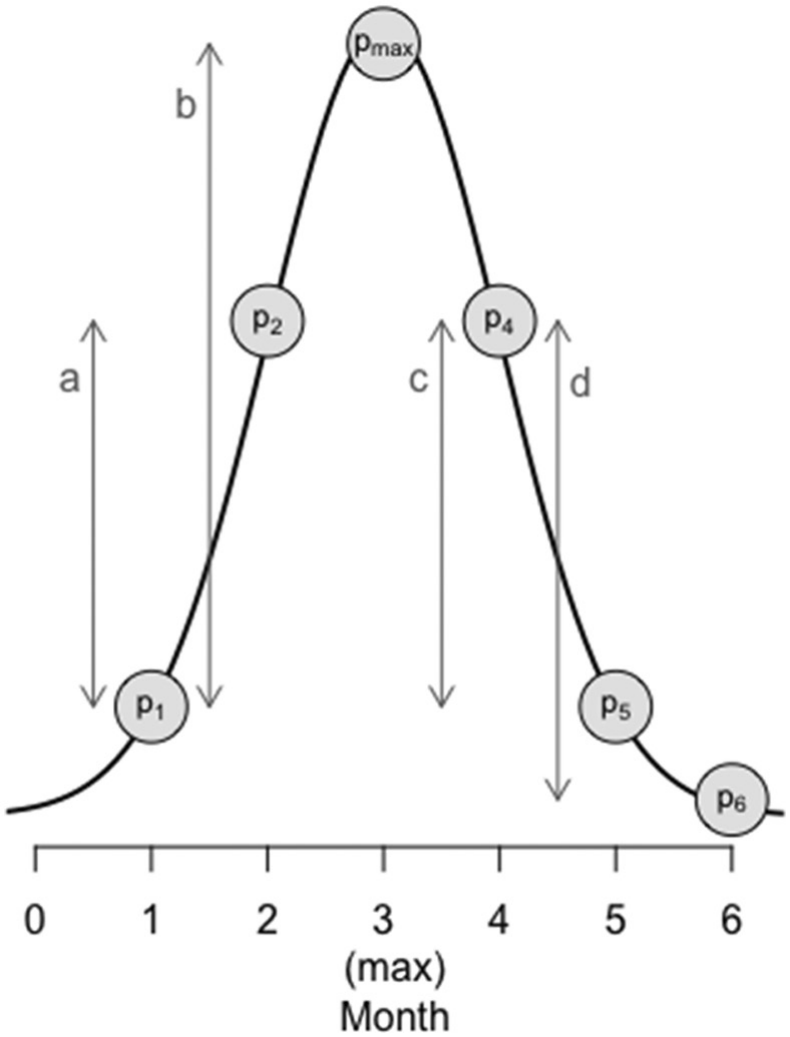

Prevalence ratio (*θ*_*t*_) is defined as

$$\theta_{t}=\left\{\begin{array}{c} \frac{p_{t+1}-p_{t}}{p_{\max}-p_{t}}\;\text{if}\;t\leq t+1\\ 1-\frac{p_{t}-p_{t+1}}{p_{t}}\;\text{if}\;t\geq t+1 \end{array}\right.$$

where *t* and *t* + 1 identify pairs of adjacent months. Prevalence in months *t*, *t* + 1, and the month (max) corresponding to peak prevalence, are denoted by *p*_*t*_, *p*_*t* + 1_, and *p*_max_, respectively.

In the illustrated example, in month 1, prevalence increases by “a” units out of a possible “b,” and so *θ*_1_ = a/b. In month 4, prevalence decreases by “c” units out of a possible “d”, and so *θ*_4_ = 1−c/d.

### 2.3 Transmission model with abiotic and biotic drivers of transmission

Ranaviruses can infect a wide range of amphibian hosts and infectious periods can range from a few days up to weeks (Gray et al., 2009). Transmission can occur both directly and indirectly, and exposure appears to induce an adaptive immune response in surviving hosts (Maniero et al., 2006). Accordingly, we modeled a host community using an SIRV framework for each species, where S, I, and R represent the number of susceptible, infectious, and recovered animals, and V represents the concentration of a free‐living infectious virion stage (Appendix S1.1). For model tractability, we limited the community to two host types that could vary in key traits including abundance and competence. As well as facilitating model analysis, this choice also reflects the empirical observation that viral loads of host species are bimodal in which there are effectively two groups of host species, one with relatively low viral loads and one with much higher viral loads (Figure S1). We included environmental transmission, whereby infectious host individuals shed the virus into the environment where it persists for some finite time and can cause new infections without host‐to‐host contact (Gray et al., 2009). We included host demography via a constant birth rate and a constant per capita mortality rate, resulting in a disease‐free equilibrium for each host species given by the ratio of the birth rate and mortality rate. For the model to reflect the viral load‐based definition of host competence, infectivity, but not susceptibility, varied between the high‐ and low‐competence hosts. This means that the rate of transmission from infectious individuals did not depend on whether transmission was to an intra‐ or interspecific host but rather on the high‐ or low‐competence status of the infectious individual. In keeping with the assumption of homogeneous host susceptibility among species, the environmental transmission rate was the same for both species.

**FIGURE 1:**
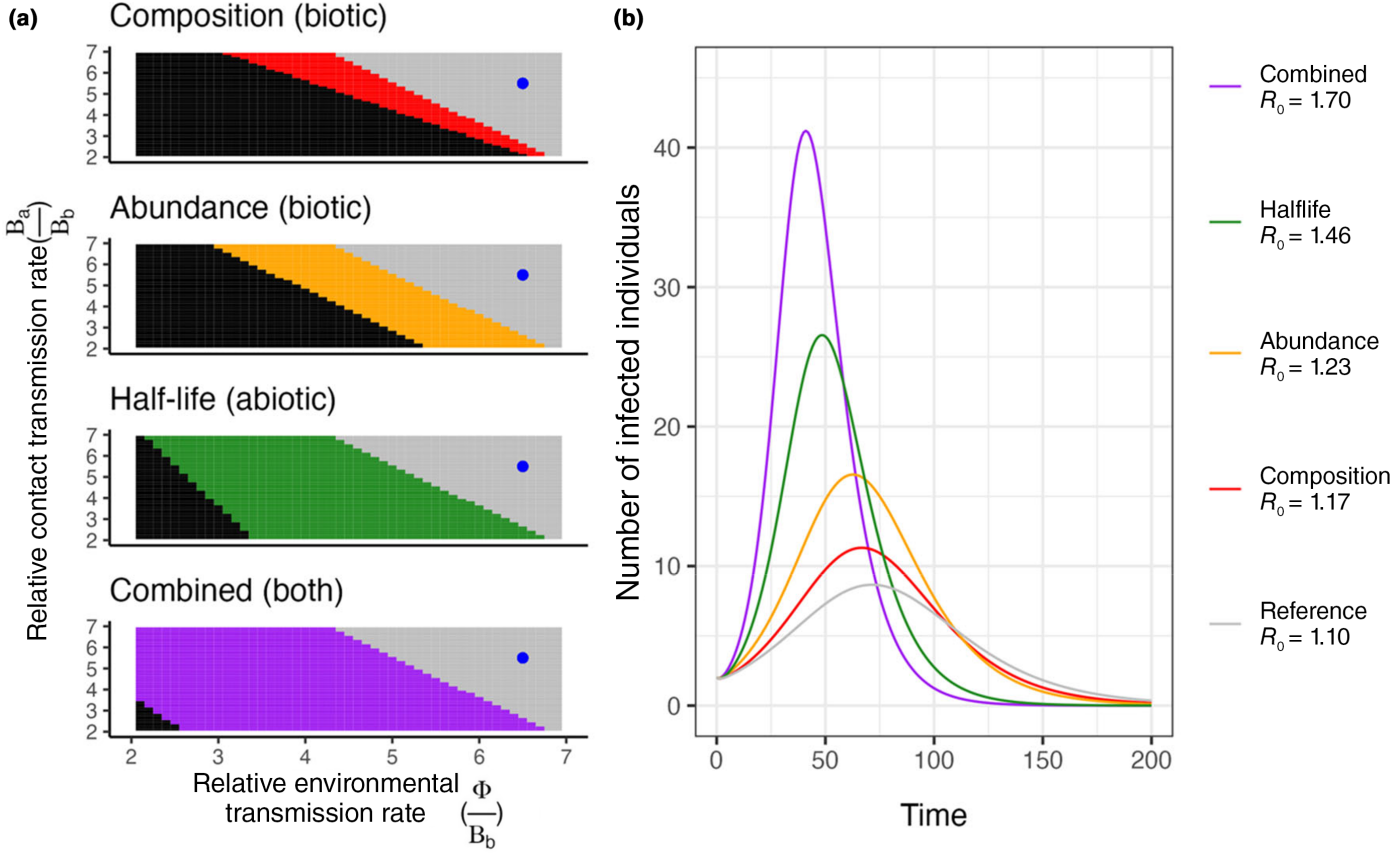
(a) Threshold of invasion under different conditions and (b) corresponding transmission dynamics. (a) The parameter space in which *R*_0_ > 1 can be increased through changes in only community composition (“Composition”, red), total host abundance (“Abundance”, gold), viral half‐life (“Half‐life”, green), or all three (“Combined”, purple). Panel labels include whether the factor is meant to represent a “biotic” or “abiotic” factor or “both”. Axes represent the ratio between either the more competent host species (β_a_) or the environmental transmission rate (ϕ) compared with the transmission rate of the less competent host species (β_b_). The gray area represents the conditions that allow for both the reference and the manipulated community to support a pathogen invasion (i.e., *R*_0_ > 1), whereas the colored areas represent the conditions where only the manipulated communities (and not the reference community) can support pathogen invasion. The black area represents the conditions that allow for neither the reference or the manipulated community to support a pathogen invasion. (b) Using the same initial conditions from the reference and manipulated communities in panel A and parameter values (blue dot) that allow for *R*_0_ to be >1, the simulated dynamics of the system show peaks with varying amplitude and timing.

Using the next‐generation matrix method (Diekmann et al., 2009), we calculated the community basic reproductive number (Dobson, 2004), hereafter referred to as *R*_0_, for our community of hosts to determine the conditions necessary for pathogen invasion (*R*_0_ > 1). Consequently, we determined how the boundary *R*_0_ = 1 is shaped as a function of parameters for communities with varying characteristics, specifically community composition, total host abundance, and viral half‐life. Guided by data (Brunner & Yarber, 2018; Gray et al., 2009; Maniero et al., 2006; Miller et al., 2011), that illustrates ranavirus transmission is promoted by biotic (large and competent host communities) and abiotic features (low temperatures), we created a reference community and four distinctly manipulated communities each designed to facilitate pathogen invasion and represent reasonable parameter estimates for this system (Appendix S1.4). The reference community had an equal number of both species, a total host abundance of 150 individuals, and a viral half‐life of 1.35 days. Viral half‐life was calculated as $\text{half}-\text{life}=\frac{\ln\left(2\right)}{\text{degradation rate}}$. Then (i) the composition‐manipulated community was altered to be dominated by the more competent species by a ratio of 2:1; (ii) the abundance‐manipulated community was altered only in abundance, to 175 individuals; and (iii) the half‐life‐manipulated community was altered by doubling the viral half‐life to 2.7 days. Finally, (iv) we constructed a manipulated community that combined each of these single‐factor manipulations. For each community, we calculated *R*_0_ over a range of values for environmental transmission rates (ϕ) and contact transmission rates of the more competent species (β_a_) while holding the contact transmission rate for the less competent species (β_b_) constant (Figure 1). This allowed us to characterize the extent to which pathogen invasion was more likely, that is, occurring for an increased set of transmission parameters that included combinations previously associated with failure to invade (*R*_0_ < 1). In addition, we observed the dynamics of these systems by numerically solving them over time to identify when peaks occurred and how high infection levels were at those peaks.

### 2.4 Patterns of community competence, host abundance, and temperature in ephemeral wetlands

Community competence, host abundance, and water temperature are all expected to influence pathogen transmission, and each of these factors varies over time and space. Community competence is fundamentally driven by the composition of hosts in the community, and to understand which hosts may be driving transmission, we ordered site‐months according to community competence values and examined which host species made up these communities. We also recorded phylogenetic distances between species to characterize how competence, as a trait, was distributed among hosts as a function of their relatedness, using a previously published phylogeny to estimate phylogenetic distances (Pyron & Wiens, 2013). To determine if there was evidence of limiting similarity or environmental filtering in host communities (Mayfield & Levine, 2010; Violle et al., 2011), we examined the relative abundance of each host in each community compared with the phylogenetic distance between that host and its closest relative in that community. If this phylogenetic distance is small between host species, this can indicate the potential for strong interspecific competition based on niche overlap, and this may reduce the abundance of each species (Webb et al., 2002; Weinstein et al., 2017). In contrast, if the phylogenetic distance is high between host species, then this may indicate that one species is ecologically distinct from others and unlikely to co‐occur in high abundance due to an environmental filtering effect. If a host species is neither phylogenetically clustered with others nor an outlier (i.e., it has a moderate phylogenetic distance to other species), then it may attain high relative abundance by avoiding both phylogenetic repulsion and environmental filtering. Finally, we measure the correlation between community competence and both host abundance and mean water temperature using Spearman rank correlation tests with Holm‐Bonferroni corrections for multiple comparisons. Correlations between these variables can be used to estimate how they covary over time and space, which can help anticipate their potential to jointly contribute to high pathogen transmission.

## 3 RESULTS

### 3.1 Effects of composition, abundance, and temperature on ranavirus transmission

Abiotic and biotic features, namely, host community composition, host abundance, and mean water temperature varied across space and time. When analyzing the relationship between these factors and relative changes in infection prevalence (prevalence ratio *θ*), community competence and host abundance both exhibit significant positive correlations with the prevalence ratio (Table 1; Figure S2a) while water temperature has a significant negative correlation. Importantly, most wetlands exhibit discernable outbreak dynamics which vary in terms of their timing and outbreak size (Figure S2b). Furthermore, patterns between community composition, abundance, and water temperature show that certain times and locations may exhibit “perfect storms” in which separate factors that moderately promote transmission (high community competence, high abundance, and lower water temperature resulting in lower rates of viral degradation) co‐occur to have larger effects (Figures S3 and S4).

**TABLE 1:** Correlations between prevalence ratio and community competence, community size, and mean water temperature.

Predictor variable	Spearman's rho	Adjusted *p*‐value
Community competence	0.478	<.001
Log_10_ (host abundance)	0.360	<.01
Mean water temperature	−0.303	<.02

*Note*: Community competence and host abundance correlated positively with prevalence ratio while mean water temperature correlated negatively.

### 3.2 Transmission model with abiotic and biotic drivers of transmission

In the transmission model, community composition, host abundance, and viral half‐life are all important promoters of transmission, and their effects are enhanced when combined. The model was used to generate demarcated transmission parameter spaces in which the pathogen is able or unable to invade and persist. This analysis was repeated to compare a baseline scenario to scenarios with improved conditions for transmission in terms of host community composition, overall host community abundance, and the persistence time of pathogen in the environment (i.e., viral half‐life). Manipulating each factor in favor of transmission (composition, abundance, and half‐life) increases the range of transmission rates that allow pathogen invasion of the host community (Figure 1a, from black zone only to black zone + colored band). However, the effect of each factor varies in the extent to which it permits invasion via lowered environmental transmission vs. contact transmission (Figure 1a). Changes in community composition result in a community that is more responsive to changes in contact transmission, that is, prone to epizootics with lower contact transmission rates. Conversely, an increase in viral half‐life renders the community more responsive to changes in environmental transmission. Abundance has an equal effect on both modes of transmission and the combined effect of all three transmission promoters results in an increase in parameter space that is appreciably greater than any individual factor alone. When observing the dynamics of these communities over time, each factor causes epizootics to occur earlier and with higher intensity compared with the reference (Figure 1b).

### 3.3 Patterns of community competence, host abundance, and temperature in ephemeral wetlands

**FIGURE 2:**
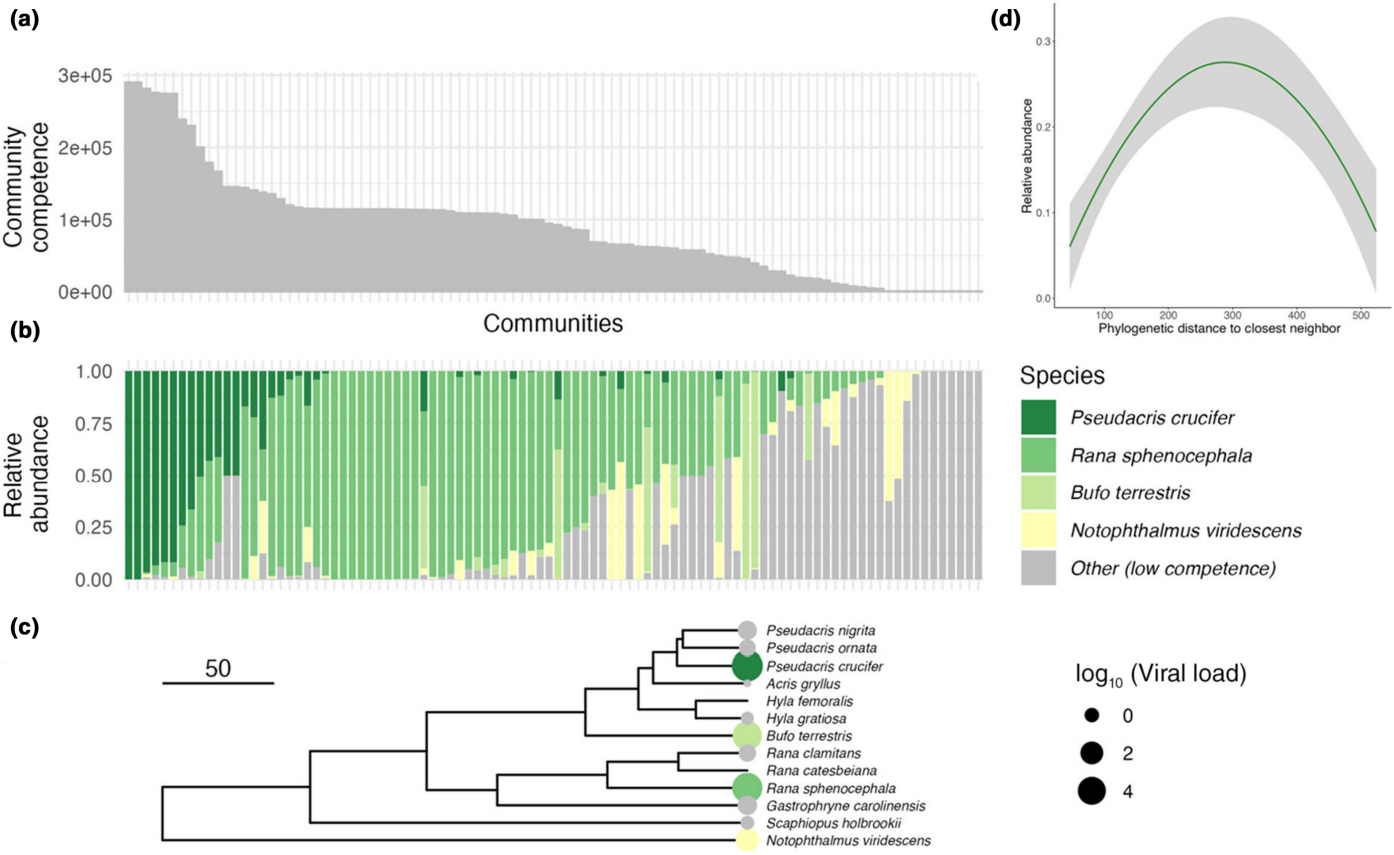
Relative abundance of host species and patterns in community competence and phylogeny. (a) All communities (site‐month combinations) were ordered according to community competence (bar lengths represent raw community competence values) and compared with (b) the relative abundance of high‐competence species (bars are filled according to the relative abundance of host species in each community). (c) The phylogeny shows that high‐competence species are moderately dispersed, suggesting that these species may not be excluded by limiting similarity in these communities. Scale bar units are in millions of years ago. The colors represent the rank order of viral load for each species. Dark green is highest and pale yellow is lowest. Gray represents species with relatively low viral load (i.e., competence). The size of each circle is proportional to the log‐transformed viral load for each species. (d) In each community, each host species' closest neighbor according to phylogenetic distance was recorded as well as the phylogenetic distance between those species. The relative abundance of each host species was then recorded against the phylogenetic distance between a host species and its closest relative in the community to characterize the relationship between how similar a host is to its closest neighbor and its relative abundance in the community.

Throughout the study period, community competence, host abundance, and mean water temperature varied over time and space, and it was not uncommon for these conditions to combine in ways that favor ranavirus transmission. When community competence was high, it was mostly in concert with the dominance of certain high‐competence species (Figure 2). These species (*P. crucifer, R. sphenocephala, B. terrestris*) have previously been observed to be common and in high abundance in the study region (Love et al., 2016). Further, several high‐competence host species were observed to co‐occur and even co‐dominate communities (Figure 2b). The phylogenetic relationships between these species suggest that they may be dissimilar enough to avert strong interspecific competition, resulting in high relative and absolute abundance of competent hosts in these communities (Figure 2d). Such co‐existence between intermediately related species may exacerbate ranavirus transmission because the competence trait (mean viral load) appears to be dispersed in the phylogeny, versus clustered among a set of closely related host species (Figure 2c).

Finally, analysis of the relationship between community competence and both host abundance and mean water temperature shows that there are significant correlations between these variables (Figure S4, *p* < .001). Community competence correlates positively with host abundance which can result in sites with many host individuals that are, on average, highly competent. The negative correlation between community competence and mean water temperature suggests that sites of high community competence may occur when water temperatures are low, again resulting in favorable conditions for pathogen transmission.

## 4 DISCUSSION

The transmission of many generalist pathogens is driven by biotic and abiotic factors, but the joint effects of these are rarely considered together. Using a mathematical model, we demonstrate that the effects of host abundance, community composition, and pathogen persistence times in the environment can result in conditions for transmission that are considerably more favorable to the pathogen than any factor alone. In addition, we find that these factors can compensate for each other, resulting in a broad range of conditions in which a pathogen may be able to successfully invade a host community. Our analysis of empirical ranavirus data suggest multiple transmission‐promoting factors may co‐occur, and we describe how each factor is likely to affect transmission. These results emphasize the importance of the joint effects of biotic and abiotic factors on the transmission of generalist pathogens, and the associated model helps to illustrate specific mechanisms likely to manifest across many host‐pathogen systems – a topic that has been recommended more broadly in the study of diversity–disease relationships (Shaw & Civitello, 2021).

Diversity–disease research is often studied as a scale‐dependent relationship that focuses on the effects of environmental gradients at larger spatial scales and the effects of host richness at local and regional scales (Rohr et al., 2019). At the intermediate spatial scale of our study, host richness, per se, is not as informative as host evenness, because it fails to capture the relative abundance of host species that contribute to the weighted average of species‐level competence (Johnson et al., 2013). Further, a singular focus on either environmental or host factors can obscure the importance of both factors at any spatial scale. For example, in our model, we show that both community composition and environmental persistence of the pathogen can enhance transmission potential overall, and the effects of each of these promoters disproportionately favor a distinct transmission mode. Specifically, as a host community becomes dominated by more competent species, the range of contact transmission rates that permit pathogen invasion increases appreciably, whereas when conditions change to increase pathogen persistence times in the environment, then it is the range of environmental transmission rates permitting pathogen invasion that increases. Because ranavirus transmission includes contact‐based and environmental transmission (Brunner et al., 2017; Sage et al., 2019), if the strength of transmission for one mode decreases, then the threshold for invasion may still be reached if the other transmission mode is sufficiently strong. The flexibility that comes from using multiple transmission modes may be especially advantageous in a changing climate. For example, increasing global temperatures may reduce the effectiveness of routes of transmission that rely on an environmentally viable stage, such as ranavirus, whereby free‐living infectious virions may not persist as long in the environment, effectively reducing the strength of environmental transmission. Such situations may even lead to the evolution of pathogens to exploit more advantageous transmission routes (Antonovics et al., 2017). The pathogen may evolve to have stronger contact transmission, and the result of this adaptation could result in shorter but more severe epidemics when host densities are at their peak.

We found that host species with the highest competence (i.e., viral load) were often also those with the highest relative abundances in their communities, indicating a potential link between host abundance and competence. If host abundance and competence are positively correlated, then this may be important for understanding diversity–disease relationships more broadly. Indeed, the connection between host life history traits and host competence is a growing area of research within disease ecology (Downs et al., 2019; Valenzuela‐Sánchez et al., 2021). An important addition to this body of work in our system is the finding that host species that were of high competence were not clustered within a phylogeny of the host species. Rather, highly competent host species were found to be only moderately related within a phylogeny, which may enhance their ability to co‐occur in host communities by avoiding strong interspecific competition. Further, in other systems, it is plausible that either phylogenetic repulsion or environmental filtering is not evident, as they are in the ranavirus system, which would create different patterns of host species relative abundance as a function of relatedness to other species. Consequently, the extent to which the patterns observed in the ranavirus system hold true across other disease systems is a promising area for future research.

Several diseases are linked to amphibian mass mortality events including ranavirus (Green et al., 2002; Price et al., 2014), chytridiomycosis (Berger et al., 1998; Skerratt et al., 2007), and severe perkinsea infection (Isidoro‐Ayza et al., 2017). The pathogens causing these diseases tend to be generalists and are likely to be affected by host community competence, host abundance, and environmental factors. For example, chytridiomycosis is now thought to have influenced declines in over 500 species (Scheele et al., 2019). Similarly, while the effects of severe perkinsea infection are often tied to ranids (Atkinson & Savage, 2023; Davis et al., 2007), recent work suggests a much broader host range that may encompass >95% of extant frogs (Chambouvet et al., 2015; Smilansky et al., 2021). Future studies may consider whether these patterns extend beyond ranavirus into other generalist pathogens affecting amphibians.

The ranavirus–larval amphibian system represents a valuable case study among diversity–disease relationships due to pronounced variation in host competence, natural variation in community composition (distinct from the more commonly studied anthropogenically generated dynamics of host species richness), and the existence of multiple transmission routes, including environmental transmission (Bienentreu & Lesbarrères, 2020; Lesbarrères et al., 2012; Tornabene et al., 2018). It remains an open question as to how commonly community abundance, composition, and environmental conditions demonstrably interact to influence the transmission of multihost pathogens. Community competence and host abundance can be positively correlated due to tradeoffs between life history traits such as reproduction and immunity (Ostfeld et al., 2010, 2014; Valenzuela‐Sánchez et al., 2021), which suggests the potential for the ideas presented here to occur more generally. However, the effects of temperature can be idiosyncratic because temperature ranges that favor host growth and pathogen transmission may not overlap (Gehman et al., 2018). Furthermore, other environmental variables, such as pH, substrate type, and UV exposure are also likely to affect environmental transmission of ranaviruses, and these factors may be of interest in future analyses. In the ranavirus–larval amphibian system, we observed a perfect storm where community competence, host abundance, and an environmental factor combined to enhance the overall transmission potential for the pathogen.

Our analysis was constrained by certain intentional and important limitations. First, while the focus of our study was on the transmission potential of ranavirus in larval amphibian communities characterized through the basic reproductive number (*R*_0_) and observations of ranavirus epizootics, other features of the system such as disease severity (Price et al., 2019), and persistence of pathogens through multiple seasons (Hall et al., 2018) may provide insight into the joint effects of both biotic and abiotic factors on the transmission of generalist pathogens, with suitable data. Second, we use species‐specific viral load as a proxy for competence and model this as infectivity in the system, but other traits such as susceptibility and behavioral exposure risk are also important features for which data were not available. Competence may be better understood as a context‐specific phenomenon that will depend on individual‐level host traits, pathogen genotype, and the environmental conditions of the interaction (Martin et al., 2016; Merrill & Johnson, 2020). An advantage of a tightly focused definition of competence, namely, viral load, is that it allowed us to study how the trait is distributed phylogenetically among host species, whereas the consideration of the other components of competence across a phylogeny may make it difficult to assess the distribution of competence more broadly.

## 5 CONCLUSION

The community ecology of generalist infectious diseases is inherently complex, with most pathogens under considerable influence of both biotic and abiotic variables, and many exploiting both contact and environmental transmission routes to optimize their transmission. By focusing on either biotic or abiotic variables, the field has identified important patterns relating to the effects of the environment and host diversity on pathogen transmission. However, failure to include mechanisms that comprise abiotic and biotic features, and their interactions, may mask important processes and even lead to misinterpretation of patterns. This is highlighted in our study by the non‐independence of promoters of transmission and their synergistic interactions. By explicitly considering both the effects of the environment and host community composition, we can better understand the context dependencies that drive pathogen transmission and more accurately predict scenarios in which changing host communities will allow pathogens to invade and persist.

## AUTHOR CONTRIBUTIONS

**Andrew W. Park:** Conceptualization (supporting); formal analysis (supporting); methodology (supporting); writing – original draft (supporting); writing – review and editing (supporting). **Daniel C. Suh:** Conceptualization (lead); formal analysis (lead); methodology (lead); visualization (lead); writing – original draft (lead); writing – review and editing (lead). **Stacey L. Lance:** Conceptualization (supporting); data curation (lead); methodology (supporting); writing – original draft (supporting); writing – review and editing (supporting).

## CONFLICT OF INTEREST STATEMENT

The authors declare no conflict of interest.

## DISCLAIMER

This report was prepared as an account of work sponsored by an agency of the United States Government. Neither the United States Government nor any agency thereof, nor any of their employees, makes any warranty, express or implied, or assumes any legal liability or responsibility for the accuracy, completeness or usefulness of any information, apparatus, product, or process disclosed, or represents that its use would not infringe privately owned rights. Reference herein to any specific commercial product, process, or service by trade name, trademark, manufacturer, or otherwise does not necessarily constitute or imply its endorsement, recommendation, or favoring by the United States.

## Supporting information


Appendices S1–S3

## Data Availability

Data can be accessed via a private link for peer review at: https://osf.io/v782c/?view_only=7e7adc48c90d4058803eefe50be0515e.
