# Supplementary material for: Abiotic and biotic factors jointly influence the contact and environmental transmission of a generalist pathogen
Source: Ecol Evol. 2024 Aug 16;14(8):e70167. doi: 10.1002/ece3.70167 (PMC11329300; doi:10.1002/ece3.70167)
Supplement: Supplementary file 1 — Appendices S1–S3 [file ECE3-14-e70167-s001.pdf]

Supplementary Materials

"Abiotic and biotic factors jointly influence the transmission of ranavirus in larval amphibian communities"

Appendix S1: Equations and Parameters

Appendix S2: Supplementary Figures

Appendix S3: Supplementary Methods

Appendix S1: Equations and parameters

Appendix S1.1: Equations

Two species of hosts interact with each other in a community. Infections can occur through interspecific, intraspecific, or environmental transmission. The rate of transmission is determined by the infectious individual or virion. Infectious individuals of both host species shed free-living infectious virions into the environment at a constant rate.

$$\frac{dS_a}{dt} = -\beta_a S_a I_a - \beta_b S_a I_b - \phi S_a V + \lambda_a - \mu_a S_a$$

$$\frac{dS_b}{dt} = -\beta_b S_b I_b - \beta_a S_b I_a - \phi S_b V + \lambda_b - \mu_b S_b$$

$$\frac{dI_a}{dt} = \beta_a S_a I_a + \beta_b S_a I_b + \phi S_a V - \alpha_a I_a - \mu I_a$$

$$\frac{dI_b}{dt} = \beta_b S_b I_b + \beta_a S_b I_a + \phi S_b V - \alpha_b I_b - \mu I_b$$

$$\frac{dR_a}{dt} = \alpha_a I_a - \mu_a R_a$$

$$\frac{dR_b}{dt} = \alpha_b I_b - \mu_b R_b$$

$$\frac{dV}{dt} = \sigma_a I_a + \sigma_b I_b - \epsilon V$$

$$\begin{bmatrix} \frac{\beta_a \frac{\lambda_a}{\mu_a}}{\alpha_a+\mu_a} & \frac{\beta_a \frac{\lambda_b}{\mu_b}}{\alpha_b+\mu_b} & \frac{\sigma_a}{\epsilon} \\ \frac{\beta_b \frac{\lambda_a}{\mu_a}}{\alpha_a+\mu_a} & \frac{\beta_b \frac{\lambda_b}{\mu_b}}{\alpha_b+\mu_b} & \frac{\sigma_b}{\epsilon} \\ \frac{\phi \frac{\lambda_a}{\mu_a}}{\alpha_a+\mu_a} & \frac{\phi \frac{\lambda_b}{\mu_b}}{\alpha_b+\mu_b} & 0 \end{bmatrix}$$

Appendix S1.2: The next-generation matrix for this model generates a 3x3 matrix and the maximum eigenvalue of this matrix represents the basic reproductive number for the pathogen.

Appendix S1.3: Definitions of Parameters and Variables

Parameter or Variable	Definition
β_a	transmission rate for more competent host
β_b	transmission rate for less competent host
ϕ	environmental transmission rate
λ	birth rate
μ_a	death rate of more competent host
μ_b	death rate of less competent host
α	recovery rate
σ	shedding rate
ϵ	viral degradation rate
S	Susceptible individuals
I	Infectious individuals
R	Recovered individuals
V	free-living infectious virions

Appendix S1.4: Parameter values and starting conditions for each model formulation (all units are in days)

Parameter or Variable	Reference	Community	Abundance	Half-life	Combined
β_a	0.0001-0.001				
β_b	0.0001				
ϕ	0.0001-0.001				
λ	1.67				
μ_a	0.0222	0.0167	0.0190	0.0222	0.0143
μ_b	0.0222	0.0333	0.0190	0.0222	0.0286
α	0.1				
σ	0.5				
ϵ	0.5134			0.2567	
S	150		175	150	175
I	1				
R	0				
V	0				

Appendix S1.5: Parameter values used to generate model dynamics (Blue dot from Figure 1A; Dynamics from Figure 1B) (all units are in days)

Parameter or Variable	Reference	Community	Abundance	Half-life	Combined
β_a	0.00055				
β_b	0.0001				
ϕ	0.00065				
λ	1.67				
μ_a	0.0222	0.0167	0.0190	0.0222	0.0143
μ_b	0.0222	0.0333	0.0190	0.0222	0.0286
α	0.1				
σ	0.5				
ϵ	0.5134			0.2567	
S	150		175	150	175
I	1				
R	0				
V	0				

Appendix S2: Supplementary Figures

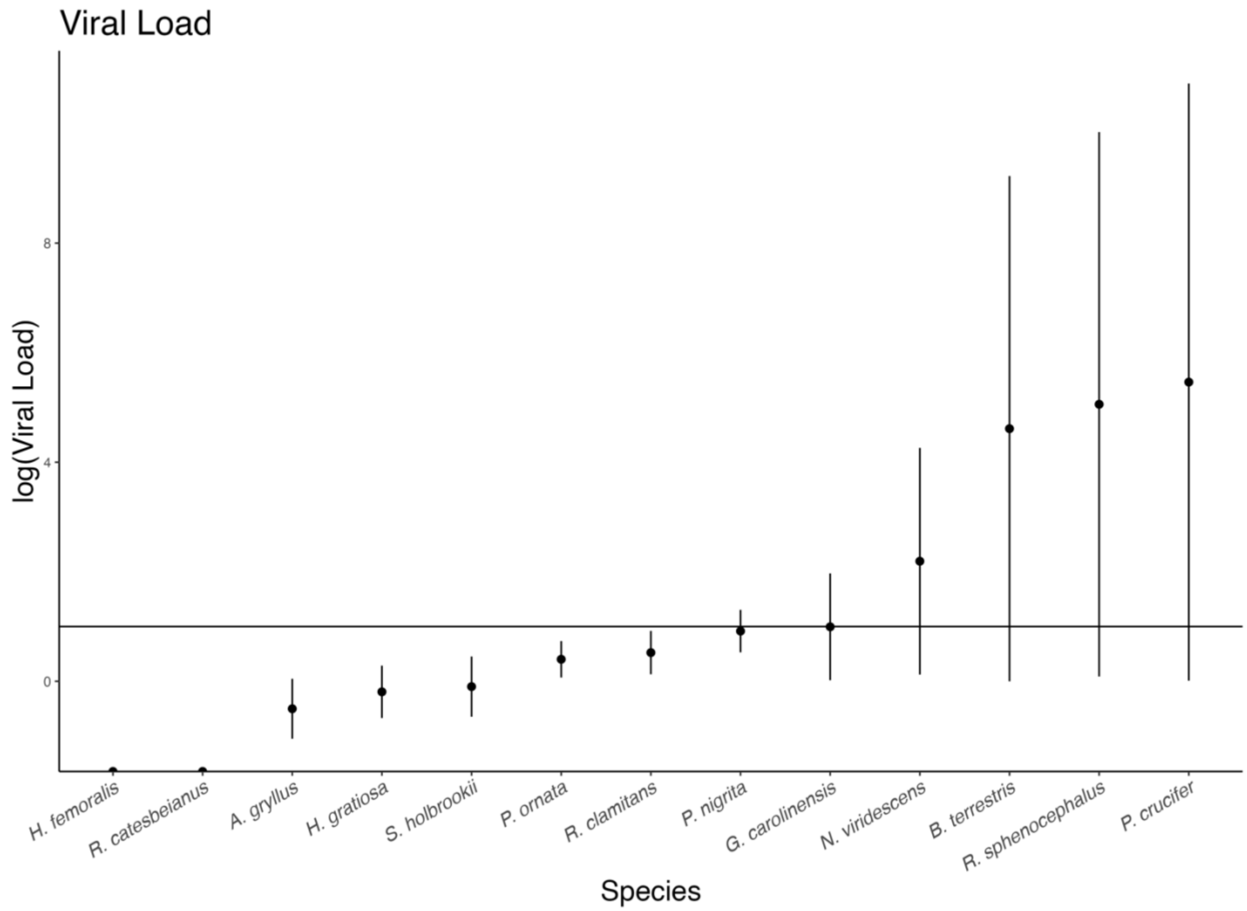

Supplementary Figure 1: Viral loads (log-transformed) of all observed host species that were sampled for ranavirus. Viral loads show a bimodal distribution where most host species have relatively low viral loads, and a few have high viral loads. Hosts above the line marking log(Viral Load) = 1 were chosen to be highlighted with green shading in Figure 2B in the main text.

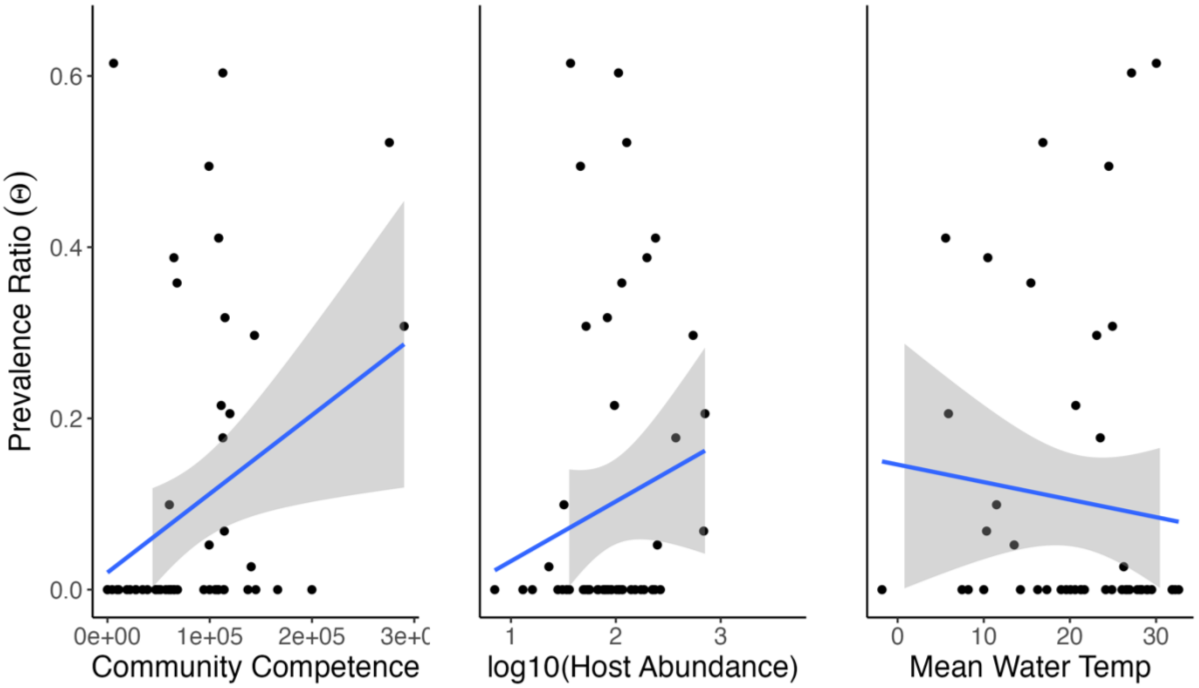

Supplementary Figure 2a: Spearman rank correlations between prevalence ratio and community competence, community size, and mean water temperature. Community competence and host abundance correlated positively with prevalence ratio while mean water temperature correlated negatively. Each point represents a single site-month combination. Correlation coefficients and associated p-values are reported in the main text in Table 1.

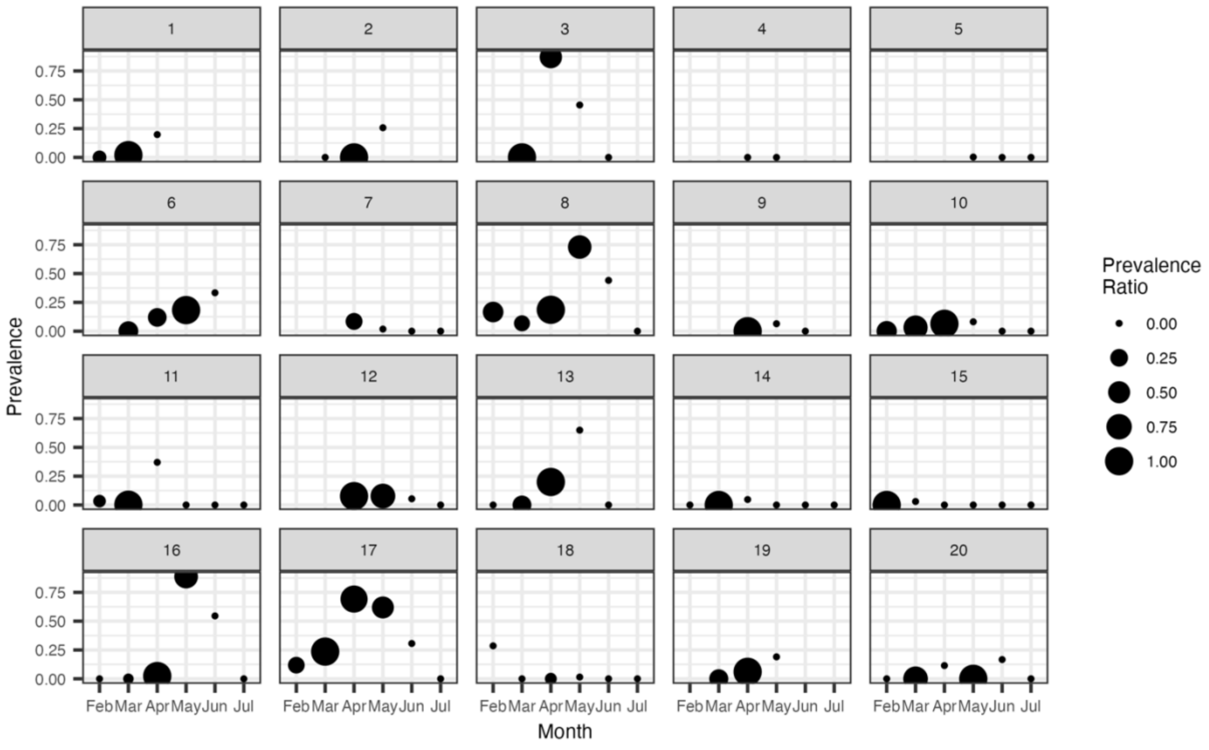

Supplementary Figure 2b: Time-series of prevalence at sampling events for each wetland. Size of points represent value of prevalence ratio. The common pattern is that sites exhibit steady increases in prevalence, peak at some prevalence value, and then steadily decrease until zero, with some sites predominantly exhibiting either the approach towards, or decline from, peak prevalence.

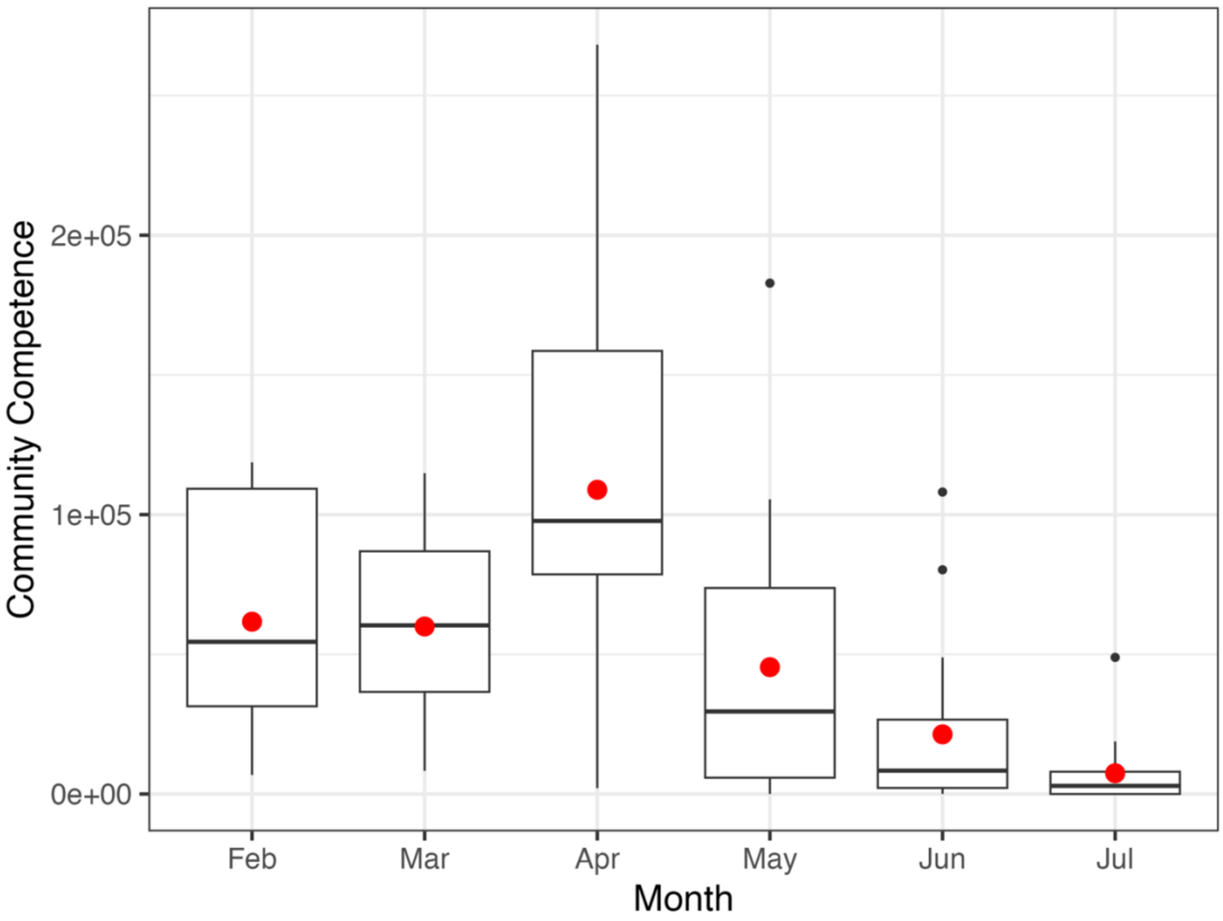

Supplementary Figure 3: Community competence of each community (site-month combination) over the duration of the study period grouped by observation month. Community competence is higher at cooler months (Feb-Mar) and peaks in April before declining in later, hotter months. Lower and upper hinges of the boxplot represent 25th and 75th percentiles, respectively, and the median is represented by the thick horizontal line within each box. Whiskers and black points represent the upper and lower ranges of the data. Average community competence across sites for each month is indicated by the red dot.

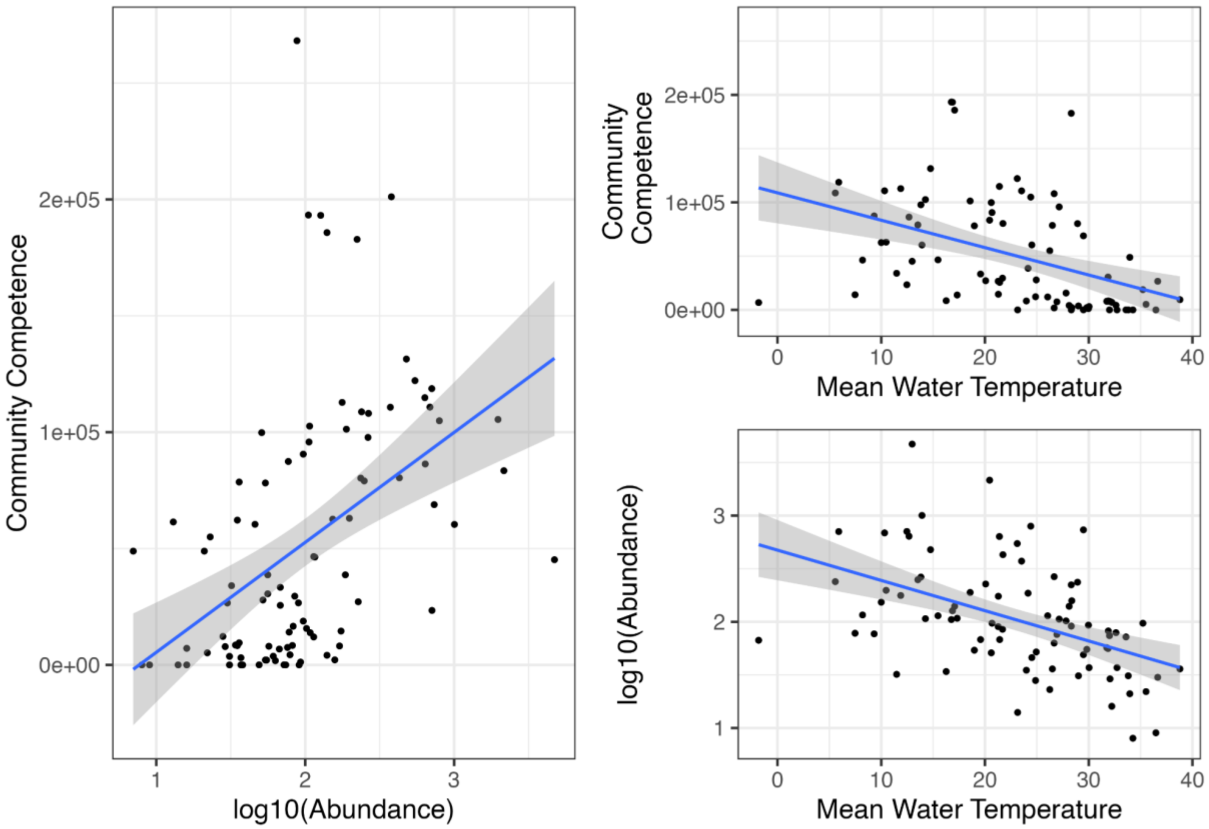

Supplementary Figure 4: Spearman rank correlations between community competence, host abundance, and mean water temperature. Community competence correlates positively with host abundance and negatively with mean water temperature. These correlations result in instances where the community has high community competence, high abundance, and low water temperature – all factors which may contribute positively to ranavirus transmission.

Appendix S3: Supplementary Methods

Weighted Prevalence Measure

Prevalence was estimated as the ratio of (a) the number of individuals found to be infected from dipnet sweeps and minnow traps from each sampling event and (b) the total number of individuals counted. However, not all individuals from dipnet sweeps and minnow traps were sampled for infection because this process required sacrificing individuals. Instead, a single individual from each host species was obtained from each dipnet sweep or minnow trap and these individuals were sampled via PCR to detect for ranavirus. To estimate prevalence at the community level (i.e., the proportion of individuals from any species that were found to be infected), we calculated the percent of ranavirus-positive individuals that were removed and sampled for ranavirus for each species and then weighed this percentage by the relative abundance of this species at each sampling event. For example, if 10% of species A sampled for ranavirus were found to be infected and species A made up 50% of the relative abundance at that sampling event, then the contribution of infected individuals from species A would be the product of 10% and 50%, i.e., 5%. This means that 5% of the total individuals at this sampling event would be considered infected individuals of species A.
